# Comparison of Intrauterine Insemination Success Rates Using Donor Sperm Between Heterosexual and Female Couples: A Retrospective Cohort Study

**DOI:** 10.5935/1518-0557.20250021

**Published:** 2025

**Authors:** Andreia Gomes, Cristina Nogueira-Silva, Pedro Brandão

**Affiliations:** 1 Escola de Medicina da Universidade do Minho, Braga, Portugal; 2 Next Fertility, Faro, Portugal

**Keywords:** female couples, fertility, heterosexual couples, intrauterine insemination, live birth rate, medically assisted reproduction

## Abstract

**Objective:**

To compare the success rates of intrauterine insemination with donated sperm between heterosexual and female couples.

**Methods:**

This was a retrospective cohort study involving 519 women who underwent intrauterine insemination with donated sperm over a 5-year period. The sample included 193 women in heterosexual relationships and 326 women in female relationships. Participants were between 18 and 38 years old, undergoing their first reproductive treatment, and had no known female fertility disorders.

**Results:**

No significant differences were found between the two groups in reproductive outcomes. Positive pregnancy test rates were 35.8% for heterosexual couples and 29.1% for female couples (*p*=0.12). Ongoing pregnancy rates were 31.6% and 27.3%, respectively (*p*=0.37). Live birth rates were 29.5% for heterosexual couples and 24.8% for female couples (*p*=0.2).

**Conclusions:**

This study found no significant differences in intrauterine insemination with donated sperm outcomes between heterosexual and female couples. These findings suggest that the absence of seminal fluid exposure in female couples may not negatively impact reproductive success. However, the role of immune tolerance in assisted reproduction outcomes remains unclear. Further multicenter studies with larger samples are needed to explore the potential influence of seminal fluid and possible clinical interventions that may replicate its effects.

## INTRODUCTION

Assisted reproductive technologies (ART) have been rapidly developing and expanding worldwide, allowing many women to achieve pregnancy. The success rates of these techniques are influenced by factors such as age, body mass index (BMI), tobacco usage, and previous obstetric history. These variables are well documented, with most studies demonstrating their implication in reproductive outcomes. They have been shown to impact success rates and often determine the necessity for additional treatment cycles in ART (Waylen *et al.*, 2008; Dondorp *et al.*, 2010; Vaegter *et al.*, 2017; Supramaniam *et al.*, 2018; Vitagliano *et al.*, 2023). Growing evidence, however, has increasingly highlighted the importance of immunologic factors in shaping these outcomes, particularly the role of exposure to seminal fluid components in reproductive success.

Bioactive signaling molecules present in semen elicit adaptive immune responses in cervical tissues after intercourse, enhancing the likelihood of successful fertilization and implantation (Bromfield, 2014). These semen-induced changes play a crucial role in facilitating the response to paternal and fetal antigens, improving receptivity to embryo implantation, and supporting fetal and placental development, ultimately influencing pregnancy success. This process is essential, as the immune system must develop tolerance to foreign antigens to accommodate the embryo and improve contact with maternal tissues during implantation (Schjenken & Robertson, 2020). Additionally, repeated exposure to the same antigens in semen can lead to the formation of memory T cells, which further enhance reproductive success in subsequent pregnancies (Robertson & Sharkey, 2016). This suggests that immune memory created by prior exposures to seminal fluid may significantly improve pregnancy outcomes, including assisted reproduction.

Empirical evidence shows that seminal fluid exposure during assisted reproduction has an immediate positive effect on pregnancy outcomes. Since 1986, studies, starting with Bellinge, have shown higher implantation rates following intravaginal plasma administration during gamete transfer, linking the presence of sperm in the reproductive tract to improved pregnancy chances. Subsequent research was done, with studies like Tremellen *et al.* (2000) further supporting the beneficial role of seminal fluid during transfer (Bellinge *et al.*, 1986; Tremellen *et al.*, 2000; Aflatoonian *et al.*, 2009; Von Wolff *et al.*, 2009, 2013; Chicea *et al.*, 2013; Friedler *et al.*, 2013). A 2014 meta-analysis reinforced these findings, showing a 23% improvement in pregnancy rates associated with seminal fluid exposure, even when individual studies lacked statistically significant results (Crawford *et al.*, 2015).

On the other hand, the role of seminal fluid in reproductive physiology suggests that the lack of exposure in homosexual women could be associated with lower pregnancy rates, a hypothesis that has not been clearly supported by the limited studies available on the subject (Nordqvist *et al.*, 2014; Brandão *et al.*, 2022).

## MATERIALS AND METHODS

This is an observational, retrospective cohort study, analyzing women who underwent intrauterine insemination (IUI) with donated sperm (D-IUI) in a private fertility clinic in Portugal between January 1st, 2019, and December 31st, 2023.

The study used a convenience, non-random sample. Women aged 18 to 38 years, in either heterosexual or homosexual relationships, and undergoing their first IUI cycle using donated sperm were included. Women with known female infertility factors were excluded. The final sample consisted of 519 patients.

Data were retrospectively gathered from medical records and anonymized before being entered into an SPSS® database for statistical analysis. Collected variables included couple type (heterosexual or female), age, body mass index (BMI), smoking habits (smoker, ex-smoker, non-smoker), type of cycle (natural or stimulated), pregnancy test results, presence of a gestational sac on ultrasound with embryonic cardiac activity, and number of live births.

Patients were divided into two groups: women in heterosexual relationships and women in female relationships. The primary outcome measured was the live birth rate, defined as the birth of a live newborn with at least 24 weeks of gestation. Secondary outcomes included positive pregnancy test rate (blood β-hCG level above the reference cutoff 15 days after insemination), ongoing pregnancy rate (detection of an embryonic heartbeat by the 8th week of gestation), miscarriage rate (non-viable pregnancy or spontaneous miscarriage), and multiple pregnancy rate (more than one live newborn).

For continuous variables, such as age and BMI, normality was assessed using visual inspection of histograms and the Kolmogorov-Smirnov test. Means and standard deviations were used to describe continuous data, while categorical variables were expressed as percentages. Statistical comparisons were made using Student’s t-tests for continuous variables and Chi-square tests for categorical variables. A significance level of 5% was set to determine statistical significance.

This study adhered to ethical principles and Good Clinical Practices. All patient data were confidential and anonymized. The study was approved by the Institutional Review Board.

## RESULTS

A total of 519 insemination cycles using donor sperm were analyzed, with patients divided into two groups: different-sex couples (n=193) and female couples (n=326).

No statistically significant differences were found in the key baseline characteristics, including age, body mass index, smoking status, and the proportion of natural or stimulated cycles (Table 1).

**Table 1:** Baseline characteristics.

		Different sex couples (n=193)	Female couples (n=326)	*p* value
Patients	Age (years; mean±SD)	32.7±4.5	32.6±3.7	0.39
	BMI (Kg/m^2^; mean±SD)	23.9±4.4)	24.1±4.5	0.57
	Smokers (n; %)	51 (27.3%)	98 (31.5%)	0.18
Cycle	Natural (n; %)	17 (8.8%)	41 (12.6%)	0.22

**Figure 1:**
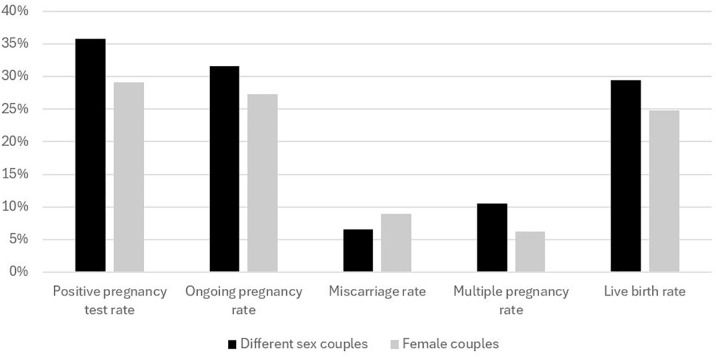
Reproductive outcomes in different-sex couples and same-sex female couples. Positive pregnancy test rates, ongoing pregnancy rates, and live birth rates were calculated based on the total number of cycles. The miscarriage rate was calculated from cases with a positive pregnancy test. The multiple pregnancy rate was calculated from all cases that resulted in a birth. None of the variables showed statistically significant differences.

Regarding obstetric outcomes, positive pregnancy tests were observed in 35.8% of different-sex couples and 29.1% of female couples (*p*=0.12). Ongoing pregnancy rates were 31.6% in different-sex couples and 27.3% in female couples (*p*=0.37). The miscarriage rate per ongoing pregnancy was 6.6% in different-sex couples and 9.0% in female couples (*p*=0.77). The live birth rate was 29.5% in different-sex couples and 24.8% in female couples (*p*=0.2). Multiple pregnancies per live birth occurred in 10.5% of different-sex couples and 6.2% of female couples (*p*=0.5) (Figure 1).

## DISCUSSION

This retrospective cohort study aimed to investigate the long-term effect of sperm exposure on reproductive outcomes by comparing fertility outcomes between female couples and different-sex couples.

Current evidence on the impact of sexual orientation on ART outcomes is mixed. Previous research suggests that sperm exposure at the time of the transfer may improve pregnancy chances (Bellinge *et al.*, 1986; Tremellen *et al.*, 2000; Crawford *et al.*, 2015). However, the available data remains limited and inconclusive.

In our study, we found no significant differences in pregnancy outcomes between different-sex couples and female couples, including positive pregnancy test rates, ongoing pregnancy rates, miscarriage rates, live birth rates, or multiple pregnancy rates. Traditionally, it has been speculated that repeated exposure to semen in heterosexual couples might affect reproductive outcomes due to potential immune responses or the development of tolerance (Bromfield, 2014; Robertson & Sharkey, 2016; Schjenken & Robertson, 2020). However, our results suggest that the absence of regular exposure to semen in female couples does not appear to negatively impact the success of IUI, raising questions about the biological importance of this factor (Nordqvist, 2014; Soares *et al*., 2019; Brandão *et al*., 2022; Dubois *et al*., 2024).

These findings are consistent with previous studies. Nordqvist *et al*. (2014) explored fertility outcomes in both different-sex and female couples, concluding that, despite the higher incidence of smoking, gynecological conditions, and other lifestyle factors among lesbian couples, no significant differences in ART outcomes were observed (Nordqvist *et al.*, 2014). This suggests that, while lifestyle factors and health conditions may differ, fertility outcomes are similar, and the absence of exposure to seminal fluid does not necessarily disadvantage their success in ART. Soares *et al*. (2019) found no significant differences in clinical outcomes of D-IUI when comparing live birth rates across heterosexual couples, single women, and lesbian couples (Soares *et al.*, 2019). This research strengthens the argument that seminal exposure, or the lack of it, does not substantially affect reproductive outcomes. Additionally, Brandão *et al*. (2022) and Dubois *et al*. (2024) both reinforce that female couples can achieve successful assisted reproduction outcomes comparable to heterosexual couples, further supporting the notion that the absence of seminal exposure does not detrimentally affect fertility outcomes (Brandão *et al.*, 2022; Dubois *et al.*, 2024).

Nevertheless, we cannot definitively conclude that prior exposure to semen is irrelevant, as we do not have data on whether female couples had previous or regular exposure to semen, just as it is not possible to be certain that different-sex couples have consistent exposure. Additionally, many heterosexual couples undergoing IUI use donor sperm due to conditions like azoospermia, meaning they may have regular exposure to semen but not to viable sperm cells.

Our study has several limitations. First, it was conducted at a single private center within one country, with a relatively small sample size, which may limit the generalizability of the findings. Additionally, we focused exclusively on IUI cycles, excluding *in vitro* fertilization (IVF) and intracytoplasmic sperm injection (ICSI) treatments. This approach was intended to minimize confounding factors, as patients pursuing IVF often present with additional infertility issues or previous failed cycles. However, it also restricts our conclusions to IUI-specific outcomes.

## CONCLUSIONS

The findings from our study did not reveal significant differences in reproductive outcomes, such as live birth rate, positive pregnancy test rate, ongoing pregnancy rate, miscarriage rate, and multiple pregnancy rate, between heterosexual and female couples undergoing intrauterine insemination with donated sperm. These results suggest that both sexual orientation and the absence of immune tolerance potentially induced by exposure to seminal fluid do not negatively impact fertility outcomes in female couples, aligning with previous studies.

However, the role of immune tolerance in assisted reproduction outcomes remains unclear. Future research, particularly multicenter studies with larger sample sizes and including other techniques such as IVF and ICSI, is needed to better understand the impact of seminal fluid exposure on assisted reproduction. This would help clarify the extent to which immune tolerance influences pregnancy outcomes and could guide new clinical interventions aimed at mimicking the potential benefits of seminal fluid exposure.
